# How Does Weight Stigma Impact Healthcare Settings? Evidence From Chilean Patients Seeking Healthcare

**DOI:** 10.1155/jobe/9983925

**Published:** 2026-09-06

**Authors:** Fernanda Bastías-González, Daniela Gómez-Pérez

**Affiliations:** ^1^ Laboratory of Stigma, Discrimination, Health, and Eating, Department of Psychology, University of La Frontera, Francisco Salazar Avenue 01445, Temuco, La Araucanía, Chile, ufro.cl; ^2^ Doctoral Program in Psychology, University of La Frontera, Avenue Francisco Salazar 01445, Temuco, La Araucanía, Chile, ufro.cl; ^3^ Nutrition and Dietetics Program, Department of Diagnostic and Evaluation Processes, Faculty of Health Sciences, Catholic University of Temuco, 56 Manuel Montt Street, Temuco, Araucanía Region, Chile

**Keywords:** healthcare providers, internalized weight stigma, medical avoidance, uncontrolled eating, weight stigma

## Abstract

**Introduction:**

Weight stigma in healthcare settings refers to the prejudiced devaluation of individuals with higher body weight, often manifested through causal attribution comments. This stigma reflects a complex interplay of psychological and behavioral processes that may contribute to outcomes such as medical avoidance.

**Objective:**

To determine whether weight stigma in healthcare settings is directly associated with internalized weight stigma, uncontrolled eating, and medical avoidance, and whether these associations are mediated through a serial pathway culminating in healthcare avoidance.

**Method:**

A cross‐sectional study was conducted with 309 healthcare users in Chile (M_age_ = 37.72 years; SD = 15.68; 75% women), using self‐report measures and structural equation modeling.

**Results:**

All direct effects were significant, except for the path between weight stigma and uncontrolled eating. The serial mediation via internalized weight stigma and uncontrolled eating was supported (*β*_indirect_ = 0.041, *p* 0.046; 12.06% of the total effect), as was the simple mediation via internalized stigma alone (*β*_indirect_ = 0.071, *p* = 0.048; 20.88%). Mediation through uncontrolled eating alone was not significant (*β*_indirect_ = 0.013, *p* = 0.239). The total effect of weight stigma in healthcare settings on medical avoidance was significant (*β*_Total_ = 0.340, *p* < 0.001; 63.24%). The model demonstrated good fit indices.

**Conclusion:**

Weight stigma in healthcare settings negatively impacts psychological and behavioral health. Addressing weight stigma is essential to reducing avoidance behaviors and improving continuity of care. These findings highlight the importance of implementing public policy reforms and providing training for healthcare professionals.

## 1. Introduction

The global prevalence of obesity continues to rise, positioning this condition as a public health priority due to its frequent association with increased cardiometabolic risk [1]. This is accompanied by disparities in the social determinants of health, as most individuals living with obesity reside in low‐ and middle‐income countries [2].

The etiopathogenesis of obesity is complex and multifactorial, resulting from a combination of factors that extend beyond excessive food intake [3]. Weight stigma, defined as negative attitudes and beliefs about a person’s weight and body size, has been linked to both the onset and perpetuation of obesity [4]. It has been conceptualized as a cyclical process, representing a process model in which individuals with higher body weight who do not conform to societal norms are judged and discriminated against, with these experiences being theoretically linked to subsequent weight gain through mechanisms such as increased consumption of palatable foods and heightened cortisol secretion [5, 6]. Weight stigma also occurs in healthcare settings [7, 8]. Although such environments should serve as safe spaces for those seeking care, evidence shows that levels of weight bias among healthcare providers are comparable to those in the general population [9]. These settings often lack the infrastructure needed to support body diversity, and healthcare providers are typically not trained to deliver inclusive care, partly due to the influence of the biomedical model that attributes excess weight to individual factors [10].

Weight stigma in healthcare settings has detrimental consequences for both psychological and physical health, often leading to maladaptive behaviors among patients [11]. Substantial evidence indicates that perceiving such stigmatization in healthcare settings is associated with poorer engagement in medical care, as individuals with higher weight frequently avoid or delay treatment due to the fear of being criticized for their body size [12]. However, medical avoidance as a maladaptive response appears to follow a more complex pathway, not fully explained by perceived weight stigma alone [13].

Internalized weight stigma represents a more proximal indicator of maladaptive behaviors than provider weight stigma. As individuals are repeatedly exposed to stigmatizing comments, they may come to believe that stereotypes, prejudices, and unfair treatment are deserved, thereby reinforcing the negative messages they encounter in clinical settings [12, 14–16].

Internalized weight stigma is linked to psychological distress and a decline in mental health, as evidenced by elevated levels of depression, anxiety, and low self‐esteem [17]. This arises from feelings of fear, guilt, shame, and body dissatisfaction, independent of body mass index (BMI). This suggests that the mechanisms of weight stigma in healthcare settings extend beyond individuals with obesity; those within the normal BMI range may also experience significant internalized weight stigma, potentially leading to disordered eating. Indeed, Remmert et al. [15] found that internalized weight stigma fully mediated the effect of stigma in healthcare settings on eating behaviors, while controlling for ethnicity, gender, and BMI.

The evidence associated with maladaptive eating behaviors is varied, though studies consistently report associations with restrictive, emotional, and uncontrolled eating [18]. Among these, emotional and uncontrolled eating are the most frequently reported in terms of significance and effect size and often co‐occur without one being more dominant than the other [4, 15, 17]. Uncontrolled eating refers to difficulty regulating food intake and may occur across a spectrum of severity, ranging from hedonic or emotionally driven overeating to the more pronounced loss of control characteristic of binge eating episodes [19].

From a cognitive–behavioral perspective, exposure to stigma can impair self‐regulation, prompting individuals to reduce or discontinue their efforts to maintain healthy eating behaviors, thereby increasing their susceptibility to disinhibited eating. This may occur when stigmatizing comments from healthcare providers are internalized and perceived as justified [15].

The loss‐of‐control component is particularly relevant among patients with cardiometabolic risk, as binge episodes are often followed by intense shame and guilt, more so than emotional eating, which does not necessarily involve excessive food intake [20]. In healthcare contexts, users frequently report uncontrolled eating as a maladaptive coping strategy in response to internalized stigma [21]. This is often linked to the belief that adopting healthy behaviors is unattainable, particularly when dietary advice is framed around body weight as the central marker of health [22]. As a result, disinhibited or uncontrolled eating may emerge as a compensatory mechanism, especially when individuals adopt all‐or‐nothing thinking and anticipate differential treatment in clinical settings. Through the internalization of weight stigma, they may come to view themselves as incapable of meeting providers’ expectations and perceive the weight‐focused discourse of physicians as unquestionable [23, 24].

A serial mediation study has examined the role of weight stigma in maladaptive eating behaviors through the internalization of weight stigma in women with postpartum depression, resulting in total mediation [25]. However, no studies to date have examined serial mediation models involving weight stigma specifically within healthcare settings. Available evidence suggests that internalized weight stigma typically follows exposure to healthcare‐based stigma, with maladaptive eating behaviors functioning as compensatory outcomes. Some studies have provided support for simple mediation, in which both internalized stigma and disordered eating mediate the link between weight stigma and medical avoidance [26, 27].

Therefore, providing evidence for a pattern of serial relationships where weight stigma in healthcare settings is associated with medical avoidance via internalization and uncontrolled eating would not only contribute to the global literature on weight stigma but would also constitute the first study to explore this mechanism in a Latin American population, a region currently underrepresented in obesity research.

Therefore, this study aims to determine whether weight stigma in healthcare settings is directly associated with medical avoidance and indirectly associated with internalized weight stigma and uncontrolled eating through a serial mediation model.

It is hypothesized that all variables will be positively associated and that two simple partial mediation pathways will emerge: (1) weight stigma in healthcare settings will influence medical avoidance via internalized stigma and (2) via uncontrolled eating. Finally, a sequential mediation is hypothesized, such that higher exposure to weight stigma in healthcare settings will predict greater medical avoidance through increased internalization and uncontrolled eating.

## 2. Method

### 2.1. Participants and Procedures

In the present cross‐sectional, correlational, and multivariate study, 309 participants were recruited using accidental nonprobabilistic sampling between March and July 2024. The target sample size was determined in accordance with methodological recommendations for structural equation modeling involving latent variables and mediation pathways, which suggest that samples of approximately 200 or more participants are generally sufficient to obtain stable parameter estimates and reliable model fit indices [28, 29]. Participation was offered remotely via the Question Pro platform or in person in healthcare institutions’ patient waiting areas and neighborhood association meetings.

Inclusion criteria required participants to be of legal age, reside in the La Araucanía Region of Chile, and have sought medical care at least once in the last 12 months. This timeframe was established to ensure that participants’ reports of healthcare‐related stigma were based on recent, lived clinical interactions, thereby reducing recall bias and ensuring the relevance of the perceived stigma in relation to current medical avoidance tendencies. Exclusion criteria included having a physical condition that limits the ability to complete the instruments and being pregnant.

Power analysis was conducted using R Studio v.2024. 12.1 with the lavaan package, which enabled Monte Carlo simulations with 1000 iterations based on standardized coefficients reported in previous studies for both direct and indirect effects [13, 17, 30, 31]. According to the hypothesized serial structural equation model (SEM), with 309 participants, the estimated statistical power was 64.4% to detect the full serial indirect effect, 82.9% for the indirect effect via internalized weight stigma, and 10.3% for the indirect effect via uncontrolled eating. Conversely, the power to detect the direct effect of providers’ weight stigma on medical avoidance was 100%. These results indicate that the sample was adequate for detecting global and simple mediation effects but only moderately powered for identifying the serial mediation effect, particularly for pathways involving uncontrolled eating.

Potential participants were invited to take part in the study trough social media, email, and flyers distributed in primary and secondary healthcare centers and neighborhood meetings. Those who agreed to participate voluntarily could choose between two formats: remotely via the “Question Pro” platform (36.56%) or in printed format at healthcare centers and neighborhood meetings (63.10%).

After reading and signing the informed consent form, which explained the study objectives, the voluntary nature of participation, and data confidentiality, participants completed a battery of self‐report instruments independently. The estimated time to complete the battery was 30 min. In the face‐to‐face modality, trained research assistants supervised this process. Participants received a summary of the main findings and educational material on active coping strategies for managing experiences of stigma in healthcare settings, which was sent to the email addresses they provided. This study was approved by the Scientific Ethics Committee of the Universidad de La Frontera (official record no. 121_23).

### 2.2. Measures

Inventory of stigmatizing situations in healthcare settings [32]: It originally consisted of 20 items to assess how often a person with high weight experienced different situations of stigmatization due to their weight in a healthcare setting in the last 12 months (e.g., “A doctor blaming unrelated physical problems on your weight”/“Un médico/a culpa a su peso de problemas físicos que no tienen relación con el peso”). The response format is scored as follows: 0 = *never*, 1 = *once*, 2 = *twice*, and 3 = *several times* in the last 12 months. Since there was no previous version in Spanish, a translation and cultural adaptation process was carried out following methodological recommendations [33, 34]. This process involved forward and back translation by three independent translators, one of whom is a native English speaker, a review by a committee of experts, and a cognitive pilot test with Chilean adults who use the public health system. This is the first study to apply the scale in Spanish. Regarding psychometric properties, preliminary analyses were conducted that confirmed the unidimensional structure, with good replicability (H index = 0.972) according to statistical recommendations [35, 36]. However, for the present study, three items (7, 15, and 20) were excluded for factor loadings < 0.40. The final version showed excellent levels of internal consistency (Cronbach’s alpha = 0.973; McDonald’s omega = 0.973).

Internalized Weight Stigma Scale (“Escala de estigma de peso internalizado [EEPI‐11]”) [37]: It contains 11 items to assess how much you agree with each statement representing internalized weight stigma (e.g., “Todo el tiempo estoy pensando que mi peso es un problema”). All items are paraphrased similarly, so that a higher score indicates greater internalized weight stigma. The response format uses a five‐point Likert scale (1 = *strongly disagree* to 5 = *strongly agree*). This scale was developed in the Chilean context, with its theoretical foundation and preliminary psychometric properties supporting its use as a measure of internalized weight stigma in the study’s target population. Its unidimensional structure was confirmed, demonstrating good replicability (H index = 0.949), and the levels of internal consistency are excellent (Cronbach’s alpha = 0.922; McDonald’s omega = 0.964).

Three‐factor eating questionnaire (TFEQ‐R21C) [38]: The uncontrolled eating subscale, consisting of nine items, was used (e.g., “A veces, cuando empiezo a comer, parece que no puedo parar”). The response format is a four‐point Likert scale ranging from 1 = *definitely false* to 4 = *definitely true*, with higher scores indicating a greater lack of control. Preliminary psychometric analyses confirmed a good model fit and adequate convergent validity using average variance extracted (AVE = 0.557), supporting the representativeness of the uncontrolled eating construct [39]. Internal consistency was excellent (Cronbach’s alpha = 0.910; McDonald’s omega = 0.893).

Medical avoidance [12]: It is composed of five items to assess avoidance of medical care after perceiving negative beliefs associated with body weight in healthcare settings. Four items have a dichotomous response format (e.g., not true/true; no/yes), and one has a Likert‐type format (e.g., 1 = *totally disagree* to 4 = *totally agree*); an example of an item is “Evito ver a mi médico/a porque siento incomodidad cuando examina mi cuerpo.”

To operationalize the variable, all items were dichotomized by coding 0 = *not/not true* and 1 = *yes/true*, and a total score was obtained by summing the responses. This decision is consistent with prior research on healthcare avoidance in the context of weight stigma, where avoidance is conceptualized as the presence or absence of a behavioral response rather than its frequency [40]. Within this framework, the occurrence of avoidance constitutes a clinically meaningful indicator, as even a single episode of avoidance or delay in care may have relevant implications for health outcomes.

A preliminary psychometric analysis revealed low interitem correlation (*r* = 0.29), which led to the exclusion of item “¿Alguna vez ha cambiado de médico/a ya que sintió que le trataba diferente debido a su peso?” The resulting scale ranged from 0 to 4, with higher scores indicating greater avoidance of medical care. Internal reliability was estimated using the Kuder–Richardson 20 (KR‐20; 0.67, considered acceptable) and McDonald’s omega (0.89, excellent), based on a polychoric correlation matrix appropriate for dichotomous items [41].

Sociodemographic questionnaire (author’s own): This questionnaire assessed gender identity, age, ethnicity, socioeconomic status (SES), self‐reported weight and height for the subsequent calculation of BMI, and dependence on health services (public or private), among other factors, to characterize the sample.

### 2.3. Data Analysis

A preliminary data analysis was conducted to detect missing values, identify outliers, and assess multivariate normality. Descriptive analyses were performed using the statistical software JASP v. 0.18.3, while the multivariate reliability and psychometric quality analyses were conducted using the lavaan, semTools, and other tools available R Studio packages [42]. A nominal alpha level of 0.05 was adopted.

For the psychometric evaluation of the instruments, confirmatory factor analyses (CFAs) were conducted to ensure appropriate structures and indicators of overall fit with their corresponding statistics in acceptable ranges [43]. Subsequently, reliability indices were estimated through internal consistency using Cronbach’s alpha and McDonald’s omega; the AVE and the H index of replicability were assessed to evaluate convergent validity and stability of the construct in the ordinal scales.

Given the dichotomous nature of the outcome variable of medical avoidance, reliability was assessed using the KR‐20 [44], McDonald’s omega, and item response theory (IRT) using a 2‐parameter logistic (2PL) model to estimate item discrimination [45].

All of these indicators provided evidence for the validity and reliability of the instruments and supported the subsequent analyses.

As a complementary analysis, BMI was included as a covariate predicting all endogenous variables; however, it was not a significant predictor and did not alter the pattern of results or model fit. Therefore, it was not retained in the final model.

Latent variables were estimated from the selected items for all constructs except for medical avoidance, which was treated as a continuous observed variable. This approach was chosen to account for measurement error and to model the underlying constructs represented by multiple questionnaire items, which is recommended when analyzing psychological constructs measured through multi‐item scales. Given the inclusion of ordinal latent variables and a continuous outcome, the DWLS estimator with robust weighted least squares with mean and variance adjusted (WLSMV) correction was used, as recommended for models involving ordinal and non‐normally distributed data [46, 47]. All factor loadings exceeded 0.40 [48].

Model fit was evaluated using conventional criteria [43], based on the chi‐square statistic (*χ* ^2^), the root mean square error of approximation (RMSEA), and the standardized root mean square residual (SRMR), as well as incremental fit indices such as the Comparative Fit Index (CFI) and the Tucker–Lewis Index (TLI). Model adequacy was determined according to the following thresholds: nonsignificant *χ* ^2^, SRMR and RMSEA < 0.080 [49], and CFI and TLI > 0.95 [43].

Finally, hypothesized indirect effects were tested, and statistical power for the proposed mediation pathways was evaluated using Monte Carlo simulations based on standardized beta coefficients reported in prior studies examining weight stigma, internalization, and eating behaviors in healthcare settings [50].

## 3. Results

The sample description analyses are presented in Table A1; the descriptive statistics and correlation analyses are detailed in Table A2. Regarding the assumption of multivariate normality, significant values were observed (Mardia’s multivariate test *p* < 0.05); however, it was unnecessary to adjust for Satorra–Bentler [51] when using the WLSMV estimator, which is appropriate for variables that do not adhere to a normal distribution.

Latent factors were estimated for experienced weight stigma in healthcare settings, internalized weight stigma, and uncontrolled eating (see Table A3). For the overall fit of the model, it was necessary to respecify the original model by adding covariances between Indicators 3 and 5 (cov = 0.470, *p* < 0.001 95% CI [0.109–0.252]) and between Indicators 11 and 16 (cov = 0.708, *p* < 0.001 95% CI [0.203–0.467]) of the latent factor of weight stigma in healthcare settings. Similarly, covariances were added between Indicators 1 and 9 (cov = 0.374, *p* < 0.001 95% CI [0.117–0.467]) of the latent factor of weight stigma internalization.

**FIGURE A1:**
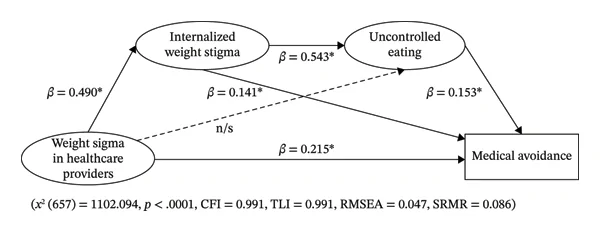
Proposed model. Note: own elaboration. ^∗^ indicates *p* < 0.001.

Consequently, a good overall fit to the data was obtained (*χ* ^2^(657) = 1102.094, *p* < 0.001, CFI = 0.991, TLI = 0.991, RMSEA = 0.047 [90% CI = 0.042–0.052], and SRMR = 086). Even though the SRMR value was slightly higher than the recommended threshold (< 0.08), the rest of the indices confirm an adequate fit to the model, considering the complexity and number of parameters (Figure A1).

Regarding the direct effects of the hypothesized model, weight stigma in healthcare settings was directly related to internalized weight stigma (*β* = 0.490, *p* < 0.001, 95% CI [0.270–0.447]) and internalized weight stigma with uncontrolled eating (*β* = 0.543, *p* < 0.001, 95% CI [0.587–0.933]). Similarly, weight stigma in healthcare settings (*β* = 0.215, *p* < 0.001, 95% CI [0.147–0.587]), internalized weight stigma (*β* = 0.145, *p* = 0.041, 95% CI [0.013–0.662]), and uncontrolled eating (*β* = 0.153, *p* = 0.034, 95% CI [0.019–0.492]) were associated with medical avoidance. We did not find a statistically significant effect of weight stigma in healthcare settings on uncontrolled eating (*β* = 0.086, *p* = 0.169, 95% CI [−0.037–0.214]).

According to the hypothesized indirect effects—notwithstanding the cross‐sectional nature of the study, which precludes establishing temporal precedence—the serial pattern of relationships yielded a small but statistically significant effect (*β*_indirect_ = 0.041, *p* = 0.046, 95% CI [0.001–0.138]), representing 12.06% of the total effect. The indirect effect via internalized weight stigma was significant (*β*_indirect_ = 0.071, *p* = 0.048, 95% CI [0.001–0.241]), implying 20.88% of the total effect; however, simple mediation via uncontrolled eating was not (*β*_indirect_ = 0.013, *p* = 0.239, 95% CI [−0.015–0.060]), accounting for 3.82% of the effect. The total effect of weight stigma in healthcare settings on medical avoidance was statistically significant (total *β* = 0.340, *p* < 0.001, 95% CI [0.357–0.803]), representing 63.24% of the effect. Given the limited statistical power for detecting the serial indirect effect, particularly for pathways involving uncontrolled eating, these results should be interpreted with caution.

## 4. Discussion

This study aimed to determine whether weight stigma in healthcare settings is directly associated with medical avoidance and indirectly linked to internalized weight stigma and uncontrolled eating through a serial mediation model. Importantly, this model is proposed as theoretical and exploratory rather than causal, given the cross‐sectional nature of the data. The results confirmed all direct effects, except for the path from weight stigma in healthcare settings to uncontrolled eating. Simple mediation via internalization and serial mediation were also confirmed regarding indirect effects. The findings demonstrate that the consequences of weight stigma in healthcare settings are multifaceted and that the mechanisms underlying these outcomes are complex. Understanding the distal phenomenon of medical avoidance requires consideration of additional psychological and behavioral mechanisms that may exacerbate avoidant behavior, as reported by several studies [26, 27, 52, 53].

Internalized weight stigma has been widely studied, and various methodologies have agreed that this internalization mediates or amplifies the consequences of weight stigma. This psychological process is associated with greater discomfort stemming from beliefs in self‐devaluation, acting as a self‐fulfilling prophecy by leading individuals to assume that unfair and hostile treatment is what they deserve from healthcare providers. This mechanism has been observed even in individuals with varying BMIs [12, 40], a finding that was also evident in this study. As Pearl and Sheynblyum [54] explain, avoiding medical settings can be a form of self‐exclusion and harm stemming from perceiving the healthcare environment as accusatory. Anticipating unfair treatment, individuals inhibit their intentions to seek medical care, thereby confirming stereotypes of irresponsibility and a lack of willpower. This cycle is perpetuated by guilt, body dissatisfaction, and low self‐esteem, leading them to believe they are incapable of adopting healthy behaviors [16].

In this study, we considered the effect of weight stigma in healthcare settings on internalized weight stigma with uncontrolled eating as a sequential mediator to medical avoidance, suggesting that internalized weight stigma may contribute to various maladaptive behaviors affecting overall health, such as engaging in uncontrolled eating behavior [25], defined by a loss of control over the amount, timing, or purpose of food intake. While this does not necessarily imply a clinical diagnosis of binge eating disorder, it does reflect impulsive eating behavior patterns [16, 55, 56]. Notably, the co‐occurrence of these eating patterns and internalized stigma has been associated with increased avoidance of healthcare services. For instance, drawing on the all‐or‐nothing theory and the belief in a just world [57], individuals may conclude that they are undeserving of health or well‐being when they fail to achieve numerical weight loss goals. Such cognitive distortions may contribute to disordered eating and reinforce the avoidance of healthcare observed in the present findings.

The serial mediation model explained 12.06% of the total effect, which is a plausible effect size considering the complexity of the processes driving avoidant behavior. Among the indirect pathways, simple mediation through internalization showed the greatest weight (20.88%) compared to the pathway that considers uncontrolled eating (3.82%). Two reasons can explain this difference. First, internalized weight stigma is one of the most studied mediating variables related to the avoidance of medical services. For example, in breast cancer screening, it has been documented that many women avoid or delay health assessments for fear of stigmatizing comments [58, 59].

Second, the statistical power to detect the indirect effect involving uncontrolled eating was low, and the power for the full serial mediation pathway was moderate. Complementary bootstrap analyses suggest that a larger sample (approximately 380 participants) would be required to achieve 80% power for detecting these effects. This limitation indicates that the observed mediation pathways, particularly those involving uncontrolled eating, should be interpreted with caution. Although the estimated effects reached statistical significance, greater emphasis should be placed on the magnitude and direction of the estimates and their confidence intervals, rather than on statistical significance alone. In this context, the role of uncontrolled eating within the serial mediation model should be interpreted considering these statistical power constraints and examined further in future studies with larger samples.

Concerning the practical implications of the results, the proposed pattern of relationships makes it clear that being treated with hostility in healthcare settings is detrimental to access to dignified healthcare and, therefore, to the lack of achievement of healthcare objectives that align with inclusive care for all people, as it will affect the continuity of health assessments and treatments [6, 60].

Interventions should focus on healthcare providers and users who are more frequently stigmatized and internalize this. For providers, interventions emphasizing continuing education on the etiology of obesity and understanding obesity as a disease [3] could help reduce the explicit attitudes of healthcare providers; weight stigma literacy and awareness of one’s own biases have proven beneficial. For example, non–weight‐focused or weight‐inclusive interventions [61] and positive affirmations that concentrate on behavioral goals have been shown to reduce the distress associated with medical avoidance [62]. For users, cognitive–behavioral interventions based on self‐compassion, acceptance, and mindfulness have proven effective in alleviating psychological distress, leading to decreased internalization, uncontrolled eating, and medical avoidance, thus contributing to increased cognitive flexibility [52, 62, 63].

Although interventions at the individual level are relevant for addressing maladaptive coping behaviors, they suggest the importance of considering structural problems in public policies, health systems, and the training of health professionals. While this study focuses on user outcomes, it is also important to recognize that the frequency of stigmatizing experiences is associated with risks associated with medical avoidance; within the context of this study, this accounted for 63.24% of the total effect. These findings point toward a potential benefit in developing antistigma health policies that regulate care practices and continuing education [64].

This study has strengths. It provides empirical evidence from a sample that has not been previously studied regarding how weight stigma operates in healthcare settings. This is particularly critical given the high prevalence of obesity in the region and the unique cultural barriers to healthcare access. The characteristics of the sample are also a strength, as they reflect a heterogeneous group in terms of age and body types, capturing perceptions primarily from the middle class and those who receive public healthcare, moving away from what is typically considered an overstudied sample (e.g., university students).

This study is not without limitations, the main one being its cross‐sectional design, which does not allow for the identification of causal relationships. Furthermore, although the instruments were rigorously translated and back‐translated, their origins in English‐speaking populations may affect their cultural relevance in the Latin American context. This difference may be even more pronounced when considering the social determinants that influence access to and choice of healthcare providers. Another limitation relates to the operationalization of the medical avoidance variable. Although the use of dichotomized items may reduce variability, this approach is consistent with prior research that conceptualizes healthcare avoidance as a behavioral outcome defined by its occurrence [40]. Future studies could incorporate measures that capture variability in the frequency or intensity of avoidance behaviors. Similarly, the sample size can be regarded as a limitation, considering the complexities of the proposed model. However, robust estimators were employed to account for data characteristics such as non‐normality and sample size, thereby improving the reliability of the parameter estimates. Furthermore, the sample was predominantly composed of women; while such a gender imbalance is common in psychological research, it nonetheless limits the generalizability of our findings to the broader population and specifically to male participants. Additionally, most participants were users of the public healthcare system, which may limit the generalizability of the findings to individuals receiving care in private or other healthcare contexts. Therefore, the results should be interpreted within the context of predominantly public healthcare users. Finally, the inclusion criterion of a medical visit within the last year may have resulted in a sample of “frequent users,” potentially excluding individuals with the highest levels of medical avoidance.

As future lines of research, we suggest confirming the proposed pattern of relationships through a longitudinal study, as this would enable us to determine causal relationships between the variables and evaluate the mediators over time. Additionally, experimental studies simulating the interaction between the provider and the user could provide background information on the implicit and explicit attitudes that generate the most stress in the user, as well as the role of self‐efficacy in users’ adherence to treatments when stigmatizing interventions are observed in comparison to weight‐inclusive interventions. Studies show that weight stigma in healthcare settings is structural and cumulative at a structural level; this involves institutional barriers and inadequate infrastructure that compound interpersonal experiences of unfair treatment by providers [22, 65, 66], and weight‐focused messages in government public campaigns to stop obesity [67], obesity control and measurement in educational institutions through the public health system [68, 69], and prevention and detection campaigns for diseases such as cancer and hypothyroidism [53, 59] would reinforce the experiences of stigmatization that have been encountered in healthcare settings, which are cumulative and contribute to medical trauma [13, 70] and to a doctor–patient relationship that is characterized by mistrust, perceived lack of empathy, and anticipation of medical stress, all of which will influence medical adherence and the perpetuation of clinical obesity [3, 16]. Consequently, future research should not only focus on measuring medical avoidance but also could further explore the doctor–patient relationship through variables such as empathy, trust in the provider, interpersonal experiences, and perception of expectations, among others.

## Author Contributions

Fernanda Bastías‐González: conceptualization, data curation, formal analysis, funding acquisition, investigation, methodology, project administration, validation, visualization, writing–original draft, and writing–review and editing.

Daniela Gómez‐Pérez: conceptualization, resources, supervision, visualization, and writing–review and editing.

## Funding

This study was funded by the Agencia Nacional de Investigación y Desarrollo, 21241321; Universidad de La Frontera, TD23‐0011.

## Conflicts of Interest

The authors declare no conflicts of interest.

## Data Availability

The data that support the findings of this study are available on request from the corresponding author. The data are not publicly available due to privacy or ethical restrictions.

## Appendix A

**TABLE A1:** Descriptive characteristics of the sample.

**Variable**	**Range**		**Mean (SD)**

Age	18–81		37.781 (15.684)
Body mass index	17.20–55.80		29.127 (6.780)

		***N***	**Percentage (%)**

Sex assigned at birth			
Female		305	98.706
Male		4	1.294
Gender identity			
Female		231	74.757
Male		75	24.272
Nonbinary		3	0.971
Sexual orientation			
Heterosexual		274	88.673
Gay		5	1.618
Bisexual		19	6.149
Other		4	1.294
Prefer not to answer		7	2.265
Chilean nationality			
Yes		306	99.029
No		3	0.971
Indigenous origin			
Not Indigenous		224	72.492
Mapuche		85	27.508
Occupation			
Student		99	32.039
Self‐employed		40	12.945
Employed		103	33.333
Unemployed		17	5.502
Retired		18	5.825
Other		32	10.356
Educational level of the highest‐income person in the household			
Incomplete primary education		12	3.883
Complete primary education		12	3.883
Incomplete secondary education		26	8.414
Complete secondary education		90	29.126
Incomplete university education		54	17.476
Complete university education		84	27.184
Postgraduate (master’s, doctorate)		31	10.032
Socioeconomic status			
Low		16	5.178
Lower middle		93	30.097
Middle		148	47.896
Upper middle		45	14.563
High		7	2.265
Health insurance			
Public (Fonasa^a^)		259	83.819
Private (Isapre^b^)		45	14.563
Semipublic (Dipreca^c^)		5	1.618
Presence of medical diagnosis			
Yes		175	56.634
No		134	43.366
Receiving treatment for medical diagnosis			
Yes		158	51.133
No		43	13.916
No diagnosis		108	34.951

^a^Fonasa is Chile’s National Health Fund, a public insurance program that finances healthcare for the country’s residents.

^b^Isapre is a health insurance institution, a private entity that offers health insurance.

^c^Dipreca is the Chilean Police Health Insurance Fund, which provides healthcare, social security, and assistance services to beneficiaries of the Chilean Police and Gendarmerie.

**TABLE A2:** Means, standard deviation, and Pearson correlations.

Variable	M	SD	1	2	3	4
1. Weight stigma among healthcare providers	0.31	0.41	—			
2. Internalized weight stigma	2.75	1.12	0.41^∗∗∗^ [0.32, 0.50]	—		
3. Uncontrolled eating	2.27	0.69	0.27^∗∗∗^ [0.16, 0.37]	0.53^∗∗∗^ [0.44, 0.60]	—	
4. Medical avoidance	1.66	1.50	0.29^∗∗∗^ [0.10, 0.39]	0.34^∗∗∗^ [0.24, 0.43]	0.31^∗∗∗^ [0.20, 0.41]	—

*Note:* The 95% confidence intervals are indicated in brackets.

^∗∗∗^ *p* < 0.001.

**TABLE A3:** Factor loadings of the proposed model.

Latent factor	Indicator	Coefficient	*p*
Weight stigma in healthcare providers	Item 1	0.783	< 0.001
	Item 2	0.844	< 0.001
	Item 3	0.826	< 0.001
	Item 4	0.8	< 0.001
	Item 5	0.73	< 0.001
	Item 6	0.837	< 0.001
	Item 8	0.785	< 0.001
	Item 9	0.903	< 0.001
	Item 10	0.835	< 0.001
	Item 11	0.73	< 0.001
	Item 12	0.762	< 0.001
	Item 13	0.844	< 0.001
	Item 14	0.804	< 0.001
	Item 16	0.722	< 0.001
	Item 17	0.858	< 0.001
	Item 18	0.689	< 0.001
	Item 19	0.856	< 0.001

Internalized weight stigma	Item 1	0.572	< 0.001
	Item 2	0.836	< 0.001
	Item 3	0.864	< 0.001
	Item 4	0.781	< 0.001
	Item 5	0.891	< 0.001
	Item 6	0.866	< 0.001
	Item 7	0.907	< 0.001
	Item 8	0.717	< 0.001
	Item 9	0.8	< 0.001
	Item 10	0.659	< 0.001
	Item 11	0.75	< 0.001

Uncontrolled eating	Item 3	0.801	< 0.001
	Item 6	0.665	< 0.001
	Item 8	0.793	< 0.001
	Item 9	0.869	< 0.001
	Item 12	0.742	< 0.001
	Item 13	0.789	< 0.001
	Item 15	0.75	< 0.001
	Item 19	0.695	< 0.001
	Item 20	0.576	< 0.001

*Note:* Confirmatory factorial analysis, factorial weights, and *p* value.
